# Optimised Degradation of Lignocelluloses by Edible Filamentous Fungi for the Efficient Biorefinery of Sugar Beet Pulp

**DOI:** 10.3390/polym16091178

**Published:** 2024-04-23

**Authors:** Zydrune Gaizauskaite, Renata Zvirdauskiene, Mantas Svazas, Loreta Basinskiene, Daiva Zadeike

**Affiliations:** 1 Department of Food Science and Technology, Faculty of Chemical Technology, Kaunas University of Technology, 50254 Kaunas, Lithuania; renata.zvirdauskiene@ktu.lt (R.Z.); loreta.basinskiene@ktu.lt (L.B.); 2 Food Institute, Kaunas University of Technology, 50254 Kaunas, Lithuania; 3 Department of Applied Economics, Finance and Accounting, Agriculture Academy of Vytautas Magnus University, 53361 Kaunas, Lithuania; mantas@svazas.lt

**Keywords:** sugar beet pulp, filamentous fungi, degradation, cellulase activity, process optimisation, biorefinery

## Abstract

The degradation of the complex structure of lignocellulosic biomass is important for its further biorefinery to value-added bioproducts. The use of effective fungal species for the optimised degradation of biomass can promote the effectiveness of the biorefinery of such raw material. In this study, the optimisation of processing parameters (temperature, time, and *s/w* ratio) for cellulase activity and reducing sugar (RS) production through the hydrolysis of sugar beet pulp (SBP) by edible filamentous fungi of *Aspergillus*, *Fusarium*, *Botrytis*, *Penicillium*, *Rhizopus*, and *Verticillium* spp. was performed. The production of RS was analysed at various solid/water (*s/w*) ratios (1:10–1:20), different incubation temperatures (20–35 °C), and processing times (60–168 h). The *Aspergillus niger* CCF 3264 and *Penicillium oxalicum* CCF 3438 strains showed the most effective carboxymethyl cellulose (CMC) degrading activity and also sugar recovery (15.9–44.8%) from SBP biomass in the one-factor experiments. Mathematical data evaluation indicated that the highest RS concentration (39.15 g/100 g d.w.) and cellulolytic activity (6.67 U/g d.w.) could be achieved using *A. niger* CCF 3264 for the degradation of SBP at 26 °C temperature with 136 h of processing time and a 1:15 solid/water ratio. This study demonstrates the potential of fungal degradation to be used for SBP biorefining.

## 1. Introduction

The growing global demand for sustainable and green technologies is promoting the development of a circular bioeconomy, where industrial waste can be transformed into higher-value products. Lignocellulosic by-products of the agricultural industry are a large resource of irrationally used biomass. The bioconversion of secondary raw materials, generated at different stages of the food production chain into bioproducts can provide a sustainable solution to renew decreasing energy resources and a rational strategy to reduce the global growth of agro-industrial waste. For the industry, it is important to apply efficient and economically useful processes for the conversion of agrobiomass into fermentable sugars, which can further be upgraded by fermentative and biocatalytic routes to value-added bioproducts [1].

Sugar beet (*Beta vulgaris* var. Saccharifera) is one of the main crops for sugar production in Europe. Approximately 280 million metric tonnes of sugar beet were produced globally in the 2019–2020 period, and the EU is the largest producer of sugar from sugar beet (approximately 46% of the world’s production) [2]. The most competitive producers in Europe are France, Germany, The Netherlands, Belgium, and Poland [3]. Lithuania is one of the largest sectors, and the sugar industry produces the most residual streams (estimated at 930,000 tons in 2023). The processing of sugar beet produces about 500,000 tons of spent sugar beet pulp (SBP) (moisture content 70–75%). Such huge quantities of biomass require reasonable implementation of a sugar beet processing by-product management system [3].

SBP, a lignocellulosic by-product of the sugar industry, was mainly used for animal feeding or as a raw material for the production of biogas [4]. Recent developments in the biotechnological valorisation of this raw material can increase its value as a raw material for the production of bioplastics, biofuels, chemicals, such as organic acids, microbial enzymes, feed proteins, and also pectic oligosaccharides [5,6].

On a dry-weight basis, SBP consists of polymeric carbohydrates (75–85% *w*/*w*), including 20–25% cellulose, 25–36% hemicelluloses, and 20–25% pectin with a low lignin content (1–3%) [7,8], and contains approximately 8% of the protein [9]. The low lignin content of SBP indicates that for the depolymerisation of this fraction, high-cost treatments are not required. The processing of biomass can be carried out by separate enzyme hydrolysis and fermentation or by simultaneous saccharification and fermentation [10]. The disadvantage of this processing might be the retardation of enzymatic hydrolysis by reaction products, which can reduce the yield of fermentable sugars released from polysaccharides. On the one hand, produced glucose and cellobiose can inhibit the activity of cellulases; however, on the other hand, the released saccharides can be consumed by microorganisms during fermentation [11]. Furthermore, cellulase production expenses in the industry are high due to the substantial enzyme loss, high energy consumption, and long processing time experienced during microbial fermentation [12]. The insolubility of cellulose leads to complex fermentation operations, making the process time and energy-consuming [13]. Even more, enzyme recovery can be largely impaired not only by the possible enzyme inactivation but also by the nonspecific and irreversible adsorption of the enzymes on the substrate, especially lignin [14]. 

Over the last decade, plant cell wall-degrading filamentous fungi have been analysed for the production of various enzymes with different catalytic activities that can be applied to the hydrolysis of renewable lignocellulosic feedstocks. Filamentous fungi are a large and diverse taxonomically group of microorganisms. They involve genera, such as *Aspergillus*, *Penicillium*, *Fusarium*, *Cladosporium*, *Emericella*, *Eurotium*, *Paecilomyces*, *Curvularia*, etc. [15]. Fungi have a unique extracellular enzyme system, including hydrolytic enzymes responsible for the degradation of various kinds of biomass polysaccharides, as well as oxidative enzymes capable of degrading lignin [16]. White-rot fungi can degrade cellulose, hemicelluloses, and lignin, whereas brown-rot fungi efficiently metabolise cellulose and hemicelluloses but can only slightly modify lignin [17]. The combination of fungal hydrolysis with other physical or chemical pretreatment methods could lead to a reduced biomass conversion time, as well as lower costs [18].

To our knowledge, there are not many reports on the optimisation of the fungal degradation-assisted production of reducing sugars in SBP. The optimisation of fermentation conditions for the production of pectinase and cellulase by *A. niger* NCIM 548 was performed on different substrates, such as wheat bran, corn bran, and kinnow peel [19]. The production of a hydrolysate of exhausted sugar beet pulp pellets as a generic microbial culture medium was carried out by Marzo et al. [20].

To develop a biotechnological pretreatment method for sugar beet pulp and its further utilisation as a medium for feed protein or bioethanol production, this study was conducted for the selection of the most active fungal strain as well as the optimisation of the processes’ parameters (temperature, time, and *s/w* ratio) for cellulase activity and reducing sugar production through the hydrolysis of SBP by edible filamentous fungi of *Aspergillus*, *Fusarium*, *Botrytis*, *Penicillium*, *Rhizopus*, and *Verticillium* spp. In this way, more valuable bioproducts can be obtained, especially in terms of sustainable biorefinery and the rational use of bioresources. 

## 2. Materials and Methods

### 2.1. Sugar Beet Pulp

Sugar beet pulp (SBP) was obtained from the company SC “Nordic Sugar Kėdainiai” (Kėdainiai, Lithuania) after saccharose extraction. The SBP material (moisture~72%) (Table 1) was frozen and stored at a −20 °C temperature. Part of the raw material was dried in a convection oven at 40 °C to a constant weight and ground using a laboratory mill (A10, IKA-Werke, Staufen, Germany) to powder (315–500 µm particles). The dried raw material was packed in tightly closed plastic bags and stored at 4 °C during the experiment. 

### 2.2. Fungal Strains

Ten fungal strains of *Aspergillus*, *Fusarium*, *Botrytis*, *Penicillium*, *Rhizopus*, and *Verticillium* spp., which were obtained from the Culture Collection of Fungi (CCF) of the Department of Botany of Charles University (Prague, Czech Republic) were used for the degradation of SBP. The fungi were preserved on a slanted potato dextrose agar (PDA, Liofilchem, Roseto degli Abruzzi, Italy) at 4 °C temperature. For the experiment, fungi were sub-cultured on the PDA slants and incubated at 25 °C, periodically sub-culturing on PDA before use.

### 2.3. Fungal Inoculum Preparation

Fungal spore suspensions were prepared from freshly sporulated (from 3- to 5-day-old) cultures according to the description by Aberkane et al. [21]. The fungal colonies were covered with 2–3 mL of a 1% Tween20 solution prepared in sterile-distilled water, and then the conidia were agitated carefully with a sterile spatula and transferred to a sterile tube. The resulting suspensions were mixed for 20 s with a vortex mixer at 2200× *g* (VWR Reax top, VWR International, Orange, CA, USA). The number of fungal spores of the inoculant was adjusted to 10^6^ CFU/mL by counting with a hematocytometer (BLAUBRAND^®^ Neubauer chamber; Merck, Madrid, Spain). 

### 2.4. Hydrolytic Activity Evaluation

The cellulose-degrading potential of ten fungal strains was determined on carboxymethyl cellulose (CMC) agar. The agar base was prepared according to Maki et al. [22], using 1% (*w/w*) of CMC. The plates were incubated at 25 °C until visible colonies were formed. Then, colonies were stained with 0.1% Congo red dye and, after 10 min, were washed off with a 1 mol/L sodium chloride solution, followed by washing with deionised water. The fungal colonies with clear zones (mm) around them were considered cellulase-active fungi. The measurements of growth colonies and hydrolysis zones were used to evaluate the hydrolytic activity expressed as a relative activity index (RAI) [23]. 

### 2.5. Shake-Flask Experiments

For the shake-flask experiment, fungal cultures were kept in 250 mL flasks with 150 mL of a specific nutrition medium containing 3 g/L of (NH_4_)_2_HPO_4_, 2 g/L of KH_2_PO_4_, 0.5 g/L of MgSO_4_, 0.5 g/L of Ca_3_(PO_4_)_2_, and 30 g/L of SBP (initial pH 5.0). Before the experiment, the medium was sterilised for 15 min at 121 °C. The tested samples were prepared by adding 1 mL of the suspension containing 10^6^ CFU/mL of the fungal spores. The samples were incubated for 7 days at 25 °C. After the first 6 and 12 h, and further after every 12 h during the 36–168 h incubation period, the production of reducing sugars was analysed.

For the one-factor experiments, the most active fungal strains (*Aspergillus niger* CCF 3264, *Fusarium solani* CCF 2967, *Botrytis cinerea* CCF 2361, *Penicillium oxalicum* CCF 3438) were selected. The test samples were prepared by mixing SBP powder (50 g) with distilled water in the glass vessels at different solid/water (*s/w*) ratios (1:10; 1:12.5; 1:15; 1:17.5; 1:20). The samples were sterilised (121 °C; 15 min) and, after cooling to 20 °C in temperature, 5 mL of the fungal spore suspension was added to each vessel. After careful mixing, the samples were incubated for 7 days at 25 °C. The second experiment was carried out at a constant *s/w* ratio (1:15) and different incubation temperatures (20, 25, 30, and 35 °C). The reducing sugar analysis was performed every 12 h during a 60–168 h incubation period. The sugar recovery was expressed as a percentage of total biomass solids, excluding protein, ash, and lignin contents. 

### 2.6. Enzyme Activity Determination

The sample extract for the enzyme activity assays was prepared according to Gasparotto et al. [24]. A homogenous SBP sample (5 ± 0.01 g) after 120 h of hydrolysis by fungi was taken and mixed with 20 mL of the 0.05 mol/L citrate buffer (pH 5). After orbital shaking (BIOSAN OS-20, SIA Biosan, Riga, Latvia) for 1 h at room temperature, the samples were centrifuged (4000× *g*; 10 min), and the obtained supernatants were collected and used as the enzyme extracts for the activity assays.

#### 2.6.1. Cellulase Activity Determination

The cellulase activity was determined according to Ghose [25], using cellulosic filter paper as the substrate. The reaction mixture (total volume 1.5 mL), containing 50 mg of filter paper strip, 0.5 mL of the sample extract, and 1 mL of sodium citrate buffer (0.05 mol/L; pH 5), was incubated for 1 h in a 50 °C water bath with shaking (120 rpm). The reducing sugars produced in the medium were determined according to Miller [26]. Control samples, consisting only of the sample extract in the buffer and the substrate in the buffer, were prepared and used to correct absorbance values. The unit of cellulase activity was defined as the amount of enzyme that released 1 µmole of glucose from 50 mg of filter paper per gram of the dry solids of the substrate per minute under assay conditions. 

#### 2.6.2. Endoglucanase Activity Determination

The endoglucanase (endo-β-1,4-glucanase) activity was determined according to Ghose [25]. For the analysis, the 0.5 mL of 1% CMC solution in 0.05 mol/L sodium acetate buffer (pH 5) was mixed with 0.5 mL of an appropriately diluted enzyme extract and incubated for 30 min at 50 °C in a water bath with shaking (120 rpm). Released reducing sugars were determined as described above. The unit of endoglucanase activity was defined as the amount of enzyme used to release 1 µmole of glucose per min from the CMC substrate under the described conditions and was expressed as U/g d.w. of SBP.

#### 2.6.3. *β*-Glucosidase Activity Determination

*β*-Glucosidase activity was determined according to the description by Verchot and Borelli [27]. The reaction mixture was prepared by mixing 0.2 mL of *p*-nitrophenyl-*β*-d-glucopyranoside (0.01 mol/L prepared in 0.05 mol/L sodium citrate buffer, pH 5) and 0.2 mL of the sample extract, which after, was incubated for 30 min in a 50 °C water bath with shaking (120 rpm). The reaction was then stopped by adding 4 mL of the 0.05 mol/L sodium hydroxide–glycine buffer (pH 10.6). The activity of *β*-glucosidase was determined by measuring the release of *p*-nitrophenol using a UV-1800 spectrophotometer (Shimadzu Corp., Kyoto, Japan) at 420 nm. The unit of *β*-glucosidase activity was defined as the amount of enzyme required to form 1 µmole of *p*-nitrophenol per minute under the assay conditions, using *p*-nitrophenyl-*β*-d-glucopyranoside as the substrate, which was expressed as U/g d.w. of SBP.

### 2.7. Chromatographic Quantification of Monosaccharides

The fermented sample (0.5 ± 0.01 g) was mixed with 5 mL of deionised water, which was ultrasonicated for 10 min (ArgoLab DU100; Chromservis s.r.o; Prague, Czech Republic), then diluted to 10 mL, cooled to room temperature, and centrifuged for 10 min at 4000× *g*. The liquid was filtered through a 0.22 µm membrane filter and used for the analysis. A High-Performance Liquid Chromatography (HPLC) system (Shimadzu Corp., Kyoto, Japan) with the ELSD detector and thermostatic column was used for arabinose, galactose, glucose, fructose, mannose, and xylose analysis. The column temperature was kept at 28 °C. The normal phase column was YMC-Pack Polyamine II (250 × 4.6 mm, I.D, 12 nm, s-5 µm) with the column guard YMC-Pack Polyamine II. The mobile phase (eluent) was a water/acetonitrile (25/75) isocratic system with a flow rate of 1 mL/min. The method’s limit of detection (LOD) was 0.5 g/L, and the limit of quantification (LOQ) was 2 g/L. The quantitative analysis was performed using calibration curves of external standards with R^2^ > 0.99. 

### 2.8. Experimental Design and Statistical Analysis

The response surface method (RSM) and central composite design (CCD) were employed for the optimisation of processing conditions for the effective production of RS and cellulase activities in the SBP substrate, evaluating the effect of selected independent variables, such as temperature (*T*, °C), processing time (*t*, min), and solid/water ration (*s/w*) at different levels. Experiments were carried out according to the three-factorial Box–Behnken design, consisting of 17 experiments and including three central points for each factor group, which were performed in random order at three replications (Table 2). Based on the preliminary investigations and data from the literature, the following conditions were adopted for the investigated process parameters that were assigned as the independent variables: *s/w* ratio (X1), *T* (X2), and *t* (X3).

The processing conditions were optimised by developing the simplest possible mathematical models with a determination coefficient higher than 80%. The responses were the RS content (Y1, g/100 g d.w.) and cellulase activity (Y2, U/g d.w.) exclusively obtained under the influence of degradation by *A. niger* CCF 3264. Equation (1) was used for the estimation of the surface response area.

Y = β_0_ + β_1_*X*1 + β_2_*X*2 + β_3_*X*3 + β_4_*X*1^2^ + β_5_*X*2^2^ + β_6_*X*3^2^ + β_7_*X*1*X*2 + β_8_*X*1*X*3 + β_9_*X*2*X*3, (1)

where βi—coefficient in the quadratic equation.

All analyses were performed at least in triplicate. The results were analysed using Minitab version 21.4.2 software (Minitab LLC, State College, PA, USA). The analysis of variance (ANOVA) was conducted for the assessment of the suitability of the mathematical models using the coefficient of ‘*lack of fit*’ and the Fisher value (*F*). Statistical analysis of the data was performed using SPSS software (ver. 27.0, IBM, Armonk, NY, USA). The significant differences between means were evaluated by the one-way ANOVA at a significance level of 0.05.

## 3. Results and Discussion

### 3.1. Selection of Potential Fungal Strains for Sugar Beet Pulp Degradation

In this study, ten strains of filamentous fungi were tested for their cellulose-degrading potential. The evaluation was based on the analysis of the ability of fungi to grow on the CMC-based agar (Figure S1) and also by comparing the relative hydrolytic activity index (RAI) values. Enzyme activities, such as cellulase, endoglucanase, and β-glucosidase, and the production of reducing sugars (RSs) were evaluated in the shake-flask experiments. 

The *Aspergillus niger*, *Penicillium oxalicum*, *Botrytis cinerea*, and *Fusarium solani* fungi were observed to have CMC hydrolytic activity with 42.8, 29.5, 61.8, and 43.2 mm, respectively, clear cellulolytic zones around their colony when plated on the CMC-based agar (Table 3). 

According to the David and Stout classification, the clear zones > 20 mm show high cellulose-degrading activity [28]. In the case of cellulose hydrolytic activity, the maximum RAI values were observed for *Penicillium oxalicum* (1.30), *Aspergillus* spp. (1.20–1.29), *Fusarium* spp. (1.14–1.16), and *Botrytis cinerea* (1.15), indicating their potential as promising cellulase producers [29]. Minimum RAI values were recorded for *Rhizoctonia solani* (1.05) and *Verticillium* spp. (1.07). 

The obtained results are consistent with Namnuch et al. [23], who demonstrated *A. flavus* KUB2 showing maximum values of the hydrolytic activity index between 1.10 and 1.12 at a 30 °C temperature, depending on the pH of the medium. 

The four most active strains were tested for cellulase, endoglucanase, and *β*-glucosidase enzyme activity production in the SBP substrate at selected conditions (25 °C; 120 h). The results of the analysis are shown in Table 4. The fungi-secreted enzyme activities in the SBP substrate varied significantly between fungal strains. 

*A. niger* CCF 3264 exhibited the highest cellulase activity (7.35 U/g d.w.), while *P. oxalicum* CCF 3438 and *B. cinerea* CCF 2361 showed similar cellulase production (5.31–5.32 U/g d.w.), while the lowest activity was determined for *F. solani* CCF 2967 (3.17 U/g d.w.). In the case of other enzyme activities, *F. solani* CCF 2967 and *P. oxalicum* CCF 3438 displayed on average 36.3–51.4% lower endoglucanase and 37.9–50.1% lower *β*-glucosidase activities compared to other strains (Table 4). Our study showed that *A. niger* CCF 3264, *P. oxalicum* CCF 3438, and *B. cinerea* CCF 2361 have the potential to produce cellulose and hemicellulose degrading enzymes.

These observations are in agreement with several reports showing the production of appropriate cellulolytic enzyme activity by several *ascomycetes* on different substrates. In the research of Vaithanomsat et al. [30], the fungus *A. niger* SOI017 was shown to be effectively producing enzyme *β*-glucosidase compared to other tested fungal strains. *A. niger* produced relatively high cellulase activity (8.89 U/g d.w.) after 72 h of incubation on Coir waste as the substrate [31], and also, the production of cellulase activity of 6.23 U/g d.w. was reported on brewery-spent grain [32]. In the study of Kumar et al., *A. niger* showed the highest cellulase activity of 10.81 U/g on the wheat bran substrate at solid-state fermentation conditions [19]. *Penicillium* sp. AKB-24, among other enzymes, produced *β*-glucosidase (6 IU/g d.s.) activity in the wheat bran substrate over 7 days of incubation at 30 °C [33]. de Oliveira Júnior et al. reused guarana processing by-products for endoglucanase (0.84 U/g) and xylanase (1.0 U/g) production by the fungus *Myceliophthora heterothallic* [34].

The adaptation of enzymatic hydrolysis for the cellulosic raw material conversion into glucose involves the synergistic hydrolysis effect on at least three different enzymes: endoglucanase, exoglucanase, and *β*-glucosidase [35]. *β*-glucosidases are the key enzymes in cellulose hydrolysis, as rate-limiting enzymes that are regulated by the feedback inhibition of the formed product of glucose, contributing to the efficiency of this process. Therefore, these enzymes are of considerable interest as constituents of cellulose-degrading systems applied to biomass conversion [36]. According to the literature, several fungal species, such as *Aspergillus*, *Fusarium*, and *Trichoderma*, can produce glucose-tolerant *β*-glucosidases [37]. Glucose-tolerant *β*-glucosidases, prevalent naturally in filamentous fungi, have a significant effect on eliminating the inhibitory effect of hexoses on alcoholic fermentation [37].

The differences in the activity of the enzymes for degrading the SBP polysaccharides were evaluated by comparing concentrations of the total RS, glucose, and arabinose that were released from SBP over 120 h of hydrolysis at 25 °C. The chromatographic analysis of saccharides showed the presence of glucose, arabinose, xylose, mannose, and fructose (Table 5).

The highest concentrations of sugars in the SBP hydrolysates were determined for arabinose, glucose, and xylose; mannose was found at slightly lower concentrations, and the lowest content was determined for fructose. The highest amounts of glucose and arabinose produced were *A. niger* CCF 3264 (5.70 and 6.57 g/100 g d.w., respectively), following *P. oxalicum* CCF 3438 (4.73 and 5.61 g/100 g d.w., respectively) and *B. cinerea* CCF 2361 (4.63–5.33 g/100 g d.w.) (Table 5).

According to the literature, relatively high glucose, arabinose, and galacturonic acid concentrations (from 12.5 to 17.0 g/L) were produced by *A. niger* AACC 11414 in the SBP hydrolysate after 166 h of incubation at a 50 °C temperature [38]. In addition, an appropriate concentration (54.8 g/L) of other saccharides, such as xylose, mannose, and galactose, was obtained [38]. In our study, the total concentration of RS produced by *A. niger* CCF 3264 in the SBP medium after 120 h of hydrolysis was 25.13 g/100 g d.w. and the total concentration of glucose and arabinose was 12.28 g/100 g d.w., corresponding to 7.90 g/L and 3.86 g/L, respectively. The lower concentration of sugars was possibly obtained due to the specificity of the strain, shorter hydrolysis time, and lower temperature. From the consistently high concentration of released arabinose, it can be concluded that almost all analysed cultures produced pectinolytic activity, even the *F. solani* culture, which had significantly lower activity. The highest glucose concentration confirmed by the highest cellulase activity (Table 4) was found after SBP hydrolysis with the *A. niger* CCF 3264 culture.

The difference in the sugar profile might be due to the different adaptations of the strains in the SBP medium and their enzyme systems. According to the literature [10], after the enzymatic hydrolysis of SBP, raffinose, arabinose, galactose, glucose, fructose, xylose, mannose, and galacturonic acid can be detected. While glucose is mainly obtained during the degradation of cellulose, pectin and hemicelluloses are providers for pentoses, such as arabinose and galactose. Hemicelluloses are also a source of xylose and mannose [39]. As the main effect on galactose release was pectinolytic activity, and it was not detected in the SBP hydrolysates, it can be assumed that the tested fungi might have had low pectinolytic activity or the process conditions were not suitable for the production of pectinases [40]. In addition, microorganisms can produce ribose when consuming xylose after the depletion of glucose. In the study of Park et al. [41], *E. coli* SGK013 were shown to produce 0.75 g/L of ribose during the batch fermentation of the medium containing glucose and xylose.

The production of different enzyme activities by fungi is highly dependent on the composition of the substrate and feedback inhibition by the hexoses. In this case, it is possible that the hydrolysis of polysaccharides and the consumption of carbon sources by fungi were more or less efficient. Moreover, the maximum catalytic activity of fungal cellulolytic enzymes, in many cases, is observed at pH 5 [42]. 

The fungal strains *A. niger* CCF 3264 and *P. oxalicum* CCF 3438, showing the highest SBP-degrading potential, except *F. solani* CCF 2967, which showed the lowest hydrolytic activity, were selected for further one-factor experiments.

### 3.2. One-Factor Experiments

**Figure 1:**
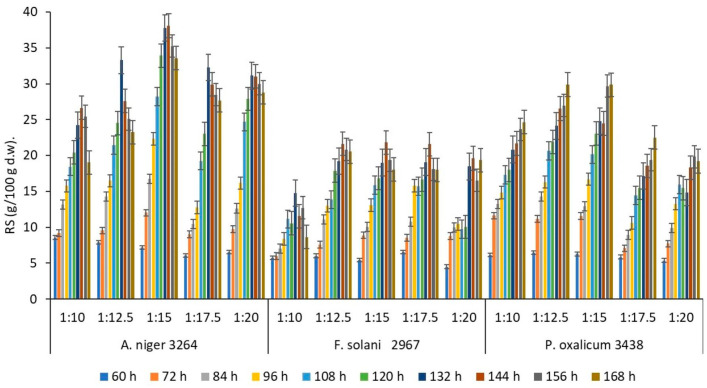
The effect of solid/water ratio (1:10–1:20) on production of reducing sugars (RSs) by selected fungal strains during 60–168 h of (25 °C, pH 5) incubation on the SBP substrate.

**Figure 2:**
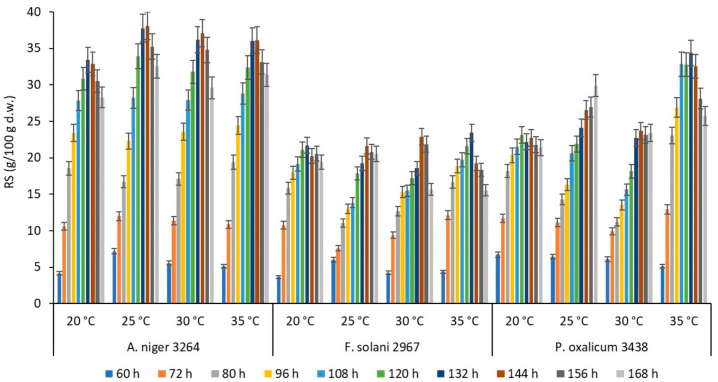
The effect of temperature on the production of reducing sugars (RSs) by selected fungal strains during 60–168 h (25 °C, pH 5) of incubation on the SBP substrate.

The one-factor experiments were carried out in order to ascertain the most active fungal strain and determine the ranges of SBP hydrolysis parameters for further process optimisation (Figure 1 and Figure 2). Factors affecting the RS production due to the fungal degradation of the SBP lignocellulose matrix were studied, measuring the amount of RS produced at different fermentation points of time and temperature (the initial RS concentration in raw material was 2.29 g/100 g d.w.).

According to the literature, temperatures between 24 and 37 °C, pH in a range of 4.0–6.5, and water activity >0.95 are optimal conditions for the growth of the most fungal strains [43,44], whereas yeast fermentation temperatures near 30 °C and pHs of 5.0–5.5 are the most suitable [45]. Thus, for the experiment, the initial pH of the medium was adjusted to 5.0 and kept constant in the case of further biomass degradation with fungi. 

As the degradation of lignocellulosic biomass is affected by moisture content, experiments on different initial solid/water ratios (*s/w*) were carried out (Figure 1). It can be seen that the RS concentration initially increased and then decreased with increasing water content. The RS concentration reached the maximum when the s*/w* ratio was varied between 1:12.5 and 1:17.5, depending on the fungal strain. Each strain showed a specific moisture optimum for maximum RS production. In the case of *F. solani* CCF 2967, the selected moisture range did not initiate significant differences in the RS contents, which depended on the duration of the process. In the case of *P. oxalicum* CCF 3438 and *A. niger* CCF 3264, the RS concentrations increased considerably, with an increasing *s/w* ratio from 1:10 to 1:15 within the 60–168 h and 60–144 h processing periods, respectively (Figure 1).

The effect of time and temperature on the RS production during the incubation of fungal strains on the SBP substrate is demonstrated in Figure 2. The *s/w* ratio was selected for each fungal strain based on the previous experiment results (Figure 1).

The highest sugar recovery rate (15.9–44.8%) was obtained at the late period of incubation (108–168 h). In most cases, processing times up to 60–80 h gave relatively low sugar recovery (3.9–27.6%). A significant increase in the RS production was observed from 96 h of processing, e.g., the highest sugar recovery was fixed for *A. niger* CCF 3264 (26.2–44.8%), following *P. oxalicum* CCF 3438 (15.9–38.2%) and *F. solani* CCF 2967 (13.3–27.6%).

In the case of processing temperature, a noticeable increase in the sugar recovery was achieved at 25–35 °C temperatures (5.0–44.8%), compared to 20 °C (4.3–39.3%) for all tested fungi, depending on the strain (Figure 2). 

The results obtained are in agreement with the reports, showing that filamentous fungi, especially basidiomycetes and ascomycetes, efficiently degrade plant lignocelluloses [46,47]. The fungi *Aspergillus niger*, *A. oryzae*, and *Trichoderma reesei* were shown to possess a variety of enzymes that could degrade the complex structure of lignocellulosic substrates [48]. Fungi, such as *A. niger*, were reported to have a flexible regulatory network adapted to the utilisation of hemicellulose and cellulose when pectin degradation was impaired [49]. In the literature, it was reported that the cultivation pH and temperature are significant factors affecting fungal hydrolytic activity [19,21]. For example, sugarcane bagasse waste samples treated by *A. flavus* KUB2 did not show cellulase activity at an incubation temperature lower than 30 °C due to the slower movement of the substrate across the fungal cells at lower temperatures, resulting in the lower yield of the products [21]. In the case of moisture content, solid-state fermentation was reported to be more effective for pectinase and cellulase production by *A. niger* compared to submerged fermentation [19]. 

The optimisation of fermentation conditions for the production of enzymes pectinase and cellulase by *A. niger* NCIM 548 was performed on different substrates, such as wheat bran, corn bran, and kinnow peel [19]. In the study of Garrigues et al., *A. niger* was involved in the SBP degradation to assess the role of pectinolytic and hemicelulolytic enzyme regulators [49]. In the work of Berlowska and co-authors, the efficiency of lactic acid production using simultaneous enzymatic hydrolysis and the fermentation of SPB was analysed [10]. Gönen and co-authors optimised the SBP acid pretreatment under pressure and non-pressure conditions [50]. 

The literature data and the obtained results of one-factor experiments demonstrated that reducing sugar production during the fungal degradation of SBP can be influenced by various processing parameters. 

### 3.3. The Optimisation of Degradation Processing and Mathematical Model Analysis

The effect of fungal hydrolysis time (*t*), temperature (*T*), and *s/w* ratio on the RS yield and cellulase activity was analysed via optimisation using CCD and RSM. The experiments were carried out according to the experimental design for three variables (Table 6), adopting the ranges of processing parameters based on the one-factor investigations (Section 3.2, Figure 1 and Figure 2). The RS yield varied significantly from 27.12 to 38.08 g/100 g d.w., and the cellulase activity values varied from 3.49 to 6.57 U/g d.w., applying different *T*, *t*, and s/w ratio combinations (Table 6).

The quartic order regression models (Table S1), describing the relationships between the RS yield (Y1), cellulase activity (Y2), and independent variables, were created in Equations (2) and (3):

Y1 = 37.909 − 0.167*X*1 + 2.150*X*2 + 0.527*X*3 − 5.152*X*1^2^ − 1.747*X*2^2^ − 1.462*X*3^2^ − 0.01*X*1*X*3 + 0.535*X*1*X*2 + 0.44*X*2*X*3, (2)

Y2 = 6.5285 − 0.0601*X*1 + 0.5148*X*2 + 0.23*X*3 − 0.6164*X*1^2^ − 0.8661*X*2^2^ − 0.6651*X*3^2^ − 0.1997*X*1*X*2 + 0.1961*X*1*X*3 − 0.0823*X*2*X*3, (3)

where Y1 is the RS yield, Y2 is cellulase activity, and *X*1, *X*2, and *X*3 are the values of the solid/water ratio, temperature, and time, respectively.

The relative errors (RE) between the experimental data and theoretical predictions varied between 0.01 and 1.26% for Y1 and between 0.08 and 1.40% for Y2 (Table 6), indicating that the models can be used to estimate the responses for the optimisation.

Based on ANOVA (Table S2), the mathematical models were significant for Y1 and Y2 and fitted well to the experimental data: Fisher values were 6.81 and 53.61 (*p* < 0.0001), and determination coefficients were R^2^ = 0.9859 and R^2^ = 0.9889 for Y1 and Y2, respectively.

The ANOVA of the models confirmed that the *s/w* ratio (*X*1) (*p* = 0.045 and *p* = 0.013), processing temperature (*X*2) (*p* = 0.0001), time (*X*3) (*p* = 0.035 and *p* = 0.0001), and their quadratic effects (*p* = 0.0001–0.005 and *p* = 0.0001) significantly affected the production of cellulase activity and RS (model *p* < 0.0001) (Table S2). The significant effects of the linear interaction between time and temperature (*X*2*X*3) (*p* = 0.049 and 0.004, respectively), and between the *s/w* ratio and temperature (*X*1*X*2) (*p* = 0.051 and *p* = 0.0001) were found. The *p*-value calculated for ‘*lack-of-fit*’ was not significant (*p* = 0.088 and 0.985, respectively); hence, the models were satisfactory in explaining the obtained data at a 95% confidence level. The predicted R^2^ values (0.9241 and 0.9699) for the experimental design are close to the adjusted R^2^ values (0.9732 and 0.9789), indicating that up to 92, and 97% of the data can be described by these regression models, respectively. 

### 3.4. Optimisation and Prediction of Process Parameters

**Figure 3:**
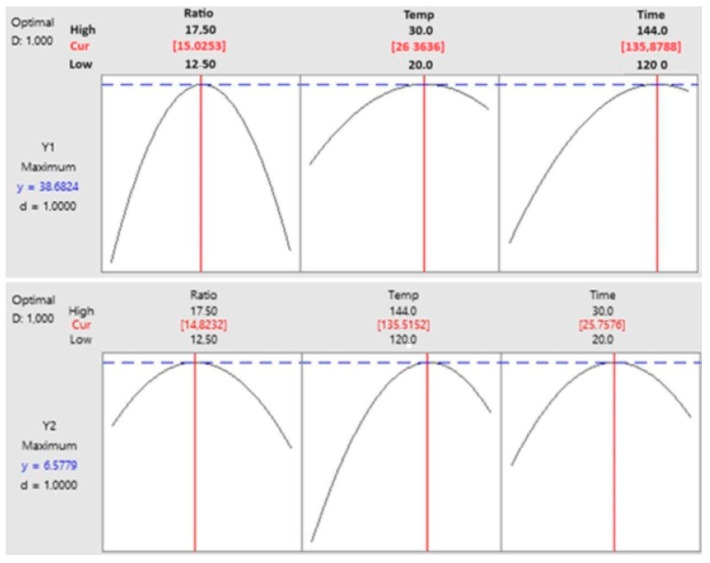
The RSM plots of the interactions between temperature and time, *s/w* and time, and *s/w* and temperature for the RS yield (Y1) and cellulase activity (Y2).

Based on the obtained mathematical models, two-dimensional plots were constructed to predict the relationship between independent variables. Predictive plots were constructed by varying the factors from the low to high level of the optimum conditions based on the increase in the RS yield or cellulase activity. Figure 3 presents the relationships between the response values (Y1 and Y2) and the independent variables (time, temperature, and *s/w* ratio). 

The elliptical contours and relatively sharp slopes in most cases of the surface responses indicate strong interactions between process parameters and a significant influence of them on the response values (Figure S2). Based on the RSM results, the optimal process conditions for the most effective production of RS (38.68 g/100 g d.w.) and cellulase activity (6.62 U/g d.w.) during the SBP degradation with *A. niger* CCF 3264 were set as follows: temperature 26 °C, time 136 h, and *s/w* ratio 1:15. 

The confirmatory experiment was performed in triplicate under the conditions suggested by mathematical models to validate them. The RS concentration was produced after 136 h of fermentation at 26 °C, the optimum *s/w* ratio was 39.15 ± 0.16 U/100 g d.w., and cellulase activity was 6.65 ± 0.08 U/g d.w. These values with RE 1.22 and 0.45%, respectively, were found to be in agreement with the RSM model’s prediction. 

In summary, the fungal degradation of biomass can be characterised as a sustainable process with the potential to produce reducing sugars from agrobiomass for further specific applications in the industry. 

## 4. Conclusions 

This study presents a concept for the biorefinery approach to pretreat and ensure the sustainable utilisation of waste sugar beet pulp biomass. An efficient, low-cost substrate and fungal strains are the key points addressed for enhanced saccharification and enzyme production at an industrial scale. The biomass after fungal hydrolysis can be used as a source of carbohydrates for yeast fermentation or microbial cultivation. Low-energy biological pretreatments overall are considered environmentally friendly processes since they do not involve chemical materials compared to most of the other pretreatments used. However, the efficiency of biological lignocellulose hydrolysis is relatively low. Thus, emerging technology, such as ultrasound, might be considered for evaluation in further experiments for biomass pretreatment in combination with fungal degradation to enhance reducing sugar yield for further applications. The results of this study provide knowledge for further research on the use of sugar beet pulp biomass for yeast fermentation or feed-value protein production, contributing to the goals of sustainable sugar beet processing.

## Figures and Tables

**Table 1:** The chemical composition (g/100 g d.w.) and total count of microorganisms of SBP.

Components	SBP
Protein	5.37 ± 0.54
Fat	0.74 ± 0.01
Sugars	6.98 ± 0.31
SDF	18.64 ± 1.22
IDF	58.65 ± 1.14
Lignin *	3.61 ± 0.35
Ash	6.01 ± 0.12
TCM	1.08 × 10^6^

* acid-insoluble lignin; SDF—soluble dietary fibre; IDF—insoluble dietary fibre; TCM—total count of microorganisms.

**Table 2:** Processing variables for the three-factorial Box–Behnken design.

Variable	Symbol	Coding Level		
		–1	0	+1
Solid/water ratio (*s*/*w*)	X1	12.5	15	17.5
Temperature (*T*, °C)	X2	20	25	30
Time (*t*, h)	X3	120	132	144

**Table 3:** Hydrolytic activity of tested fungal strains on CMC-based agar.

Fungal Strain	Clear Zone Diameter, mm	Colony Diameter, mm	RHA
*Botrytis cinerea* CCF 2361	61.84 ± 2.61	53.71 ± 1.89	1.15
*Aspergillus nidulants* CCF 2912	31.71 ± 0.93	26.34 ± 0.89	1.20
*Aspergillus niger* CCF 3264	42.78 ± 1.07	33.17 ± 1.07	1.29
*Fusarium avenaceum* CCF 3306	71.68 ± 1.84	62.81 ± 0.83	1.14
*Fusarium solani* CCF 2967	43.20 ± 5.23	37.43 ± 4.16	1.16
*Fusarium oxysporum* CCF 1389	44.88 ± 2.63	39.13 ± 1.94	1.15
*Fusarium graminearum* CCF 1626	54.33 ± 3.06	47.33 ± 1.08	1.15
*Penicillium oxalicum* CCF 3438	29.47 ± 1.53	22.67 ± 0.79	1.30
*Rhizoctonia solani* CCF 1360	43.17 ± 2.06	41.24 ± 1.79	1.05
*Verticillium* spp. CCF 1896	44.21 ± 3.17	41.07 ± 2.83	1.07

**Table 4:** Cellulolytic enzymes activities (U/g d.w.) in shake-flask experiments.

Fungi	Cellulase	Endoglucanase	β-Glucosidase
*A. niger* CCF 3264	7.35 ± 0.56 ^a^	1.72 ± 0.31 ^a^	0.77 ± 1.36 ^a^
*B. cinerea* CCF 2361	5.33 ± 0.41 ^b^	1.39 ± 0.14 ^b^	0.62 ± 1.76 ^b^
*F. solani* CCF 2967	3.17 ± 0.27 ^c^	0.94 ± 0.12 ^c^	0.31 ± 1.95 ^d^
*P. oxalicum* CCF 3438	5.31 ± 0.49 ^b^	0.83 ± 0.08 ^d^	0.46 ± 0.22 ^c^

Values are mean ± SD (*n* = 3). Different superscript letters in the same column represent significant differences at *p* < 0.05.

**Table 5:** The total reducing sugars (RSs) and monosaccharides (g/100 g d.w.) produced during the 120 h shake-flask cultivation of fungi on SBP substrate.

Fungi	Total RS	Fructose	Glucose	Arabinose	Xylose	Mannose
*A. niger* CCF 3264	25.13 ± 1.34	0.927	5.704	6.572	4.806	3.897
*B. cinerea* CCF 2361	24.86 ± 0.87	1.906	4.633	5.326	5.649	4.385
*F. solani* CCF 2967	21.06 ± 0.57	1.995	4.272	4.501	3.109	3.141
*P. oxalicum* CCF 3438	24.17 ± 0.36	1.404	4.726	5.608	5.109	4.549

Values are mean ± SD (*n* = 3).

**Table 6:** Process variables and observed responses.

Exp. No.	Process Variables			Y1 (g/100 g d.w.)			Y2 (U/g d.w.)		
	*X*1 (s/w)	*X*2 (*T*, °C)	*X*3 (*t*, h)	Exp	Predicted	/RE/(%)	Exp	Predicted	/RE/(%)
1	1	1	1	33.18 ± 1.02	33.02	0.48	4.98	4.99	0.20
2	1	1	−1	31.21 ± 0.86	31.11	0.32	4.54	4.54	0.10
3	1	−1	1	27.12 ± 0.32	26.77	1.26	4.30	4.31	0.21
4	1	−1	−1	26.63 ± 0.64	26.62	0.04	3.49	3.52	0.80
5	−1	1	1	32.33 ± 0.68	32.31	0.07	5.14	5.13	0.21
6	−1	1	−1	30.24 ± 0.37	30.35	0.37	3.85	3.86	0.49
7	−1	−1	1	28.15 ± 0.35	28.20	0.17	4.30	4.31	0.25
8	−1	−1	−1	27.68 ± 1.12	28.00	1.15	3.64	3.63	0.11
9	1	0	0	32.58 ± 0.87	32.59	0.01	5.81	5.87	1.10
10	0	1	0	37.86 ± 0.57	38.31	1.18	6.12	6.20	1.27
11	0	0	1	36.96 ± 0.51	36.97	0.04	6.13	6.11	0.25
12	−1	0	0	33.08 ± 0.33	32.92	0.47	5.91	5.99	1.40
13	0	−1	0	34.26 ± 1.02	34.01	0.73	5.15	5.17	0.37
14	0	0	−1	35.91 ± 0.28	35.92	0.01	5.59	5.65	1.13
15	0	0	0	38.06 ± 0.86	37.91	0.40	6.54	6.55	0.08
16	0	0	0	38.04 ± 1.14	37.91	0.34	6.56	6.55	0.14
17	0	0	0	37.96 ± 0.87	37.91	0.13	6.57	6.55	0.31

*T*: temperature; *t*: time; *s/w*: solid-to-water ratio; RE: relative error.

## Data Availability

Data are contained within the article and Supplementary Materials. The data presented in this article are part of first author’s PhD work.

## Supplementary Materials

The following supporting information can be downloaded at: https://www.mdpi.com/article/10.3390/polym16091178/s1, S1: Chemical and microbiological analysis; S2: Determination of soluble and insoluble dietary fibre; S3: Lignin determination; Table S1: Coded coefficients of the mathematical models for Y1 and Y2 responses; Table S2: ANOVA of mathematical models; Figure S1: Hydrolytic activity of tested fungal strains on CMC-based agar; Figure S2: Response surface plots of the interactions between process parameters for RS yield (Y1) and cellulase activity (Y2). References [51,52,53] are cited in the Supplementary Materials.
