# Supplementary material for: Optimised Degradation of Lignocelluloses by Edible Filamentous Fungi for the Efficient Biorefinery of Sugar Beet Pulp
Source: Polymers (Basel). 2024 Apr 23;16(9):1178. doi: 10.3390/polym16091178 (PMC11085495; doi:10.3390/polym16091178)
Supplement: Supplementary file 1 [file polymers-16-01178-s001.zip › polymers-2842828-supplementary.pdf]

Article

Optimised Degradation of Lignocelluloses by Edible Filamentous Fungi for the Efficient Biorefinery of Sugar Beet Pulp

Zydrune Gaizauskaite [1,2,*], Renata Zvirdauskiene [1], Mantas Svazas [3], Loreta Basinskiene [1] and Daiva Zadeike [1,*]

[1] Department of Food Science and Technology, Faculty of Chemical Technology, Kaunas University of Technology, 50254 Kaunas, Lithuania
[2] Food Institute, Kaunas University of Technology, 50254 Kaunas, Lithuania
[3] Department of Applied Economics, Finance and Accounting, Agriculture Academy of Vytautas Magnus University, 53361 Kaunas, Lithuania
* Correspondence: zydrune.gaizauskaite@ktu.lt (Z.G.); daiva.zadeike@ktu.lt (D.Z.)

Supplementary Materials

S1. Chemical analysis

The raw material was analysed for moisture, fibre, protein, sucrose, and ash contents according to the AOAC Official Methods [51]. For the moisture content determination an oven-drying method was used (drying at 135 °C for 2 h) (method 930.15). The crude protein content was determined by the Kjeldahl nitrogen (method 920.152, nitrogen to protein conversion factor was 6.25). Sucrose was determined according to the method 942.20.

S2. Determination of soluble and insoluble dietary fibre

The quantification of soluble (SDF) and insoluble (IDF)dietary fibres was measured by using an integrated total dietary fibre assay procedure (K-TDFR, Megazyme, Co. Wicklow, Ireland), including the key attributes of the validated AOAC Official Methods 2002.02, 985.29, and 991.43 [51]. Shortly, powdered SBP sample (1.00 ± 0.01 g) was mixed with MES-TRIS (2(N-morpholino)ethanesulfonic acid tris(hydroxymethyl)aminomethane) buffer (pH 8.2) and subjected to sequential enzymatic hydrolysis by heat-stable α-amylase (100 °C; 30 min). After amylase hydrolysis, sample was cooled to 60 °C and hydrolysed with protease (60 °C; 30 min). Further, the pH was adjusted to 4.8 and sample was hydrolysed with amyloglucosidase for 30 min., filtered using a vacuum through the crucible and washed twice with 60°C deionised water. The filtrate was collected and used for the SDF determination. SDF was precipitated from filtrate with 95% ethanol heated to 60 °C and recovered on the crucible by washing with distilled water, 78% ethanol, 95% ethanol, and acetone. For the determination of IDF, the sediment after enzymatic hydrolysis was then further washed with 95% ethanol and acetone. Crucibles containing residues were dried overnight in 105 °C oven and weighed. Obtained SDF and IDF contents were corrected for protein and ash. The content of each fibre was expressed as g/100 g d.w. of SBP. The analysis were performed in triplicate.

S3. Lignin determination

The determination of acid-insoluble lignin was based on the NREL methodology [52]. Some modifications have been made to the sample size and heating procedure [53]. The sample was prepared by mixing 1.0 ± 0.01 g (m_o) of the SPB sample with 15.00 ± 0.01 mL of 72% sulfuric acid and kept in a 30 °C water bath for 1 h. Then, 575 mL of deionised water were added, and the sample was boiled under reflux for 4 h. After heating, the solution was filtered through a pre-weighted fritted glass filter (m_1) and washed with boiling water until neutral pH (7.0). The neutralised filter was then oven-dried overnight at 105 °C and weighed (m_2). Insoluble lignin was calculated according to the eq.:

$$Insoluble\ lignin, \% = \frac{m_2 - m_1}{m_0} \times 100 \quad (1)$$

where: m_o – initial sample weight, g; m_1 – initial filter weight, g; m_2 – final dry weight, g.

Figures and Tables

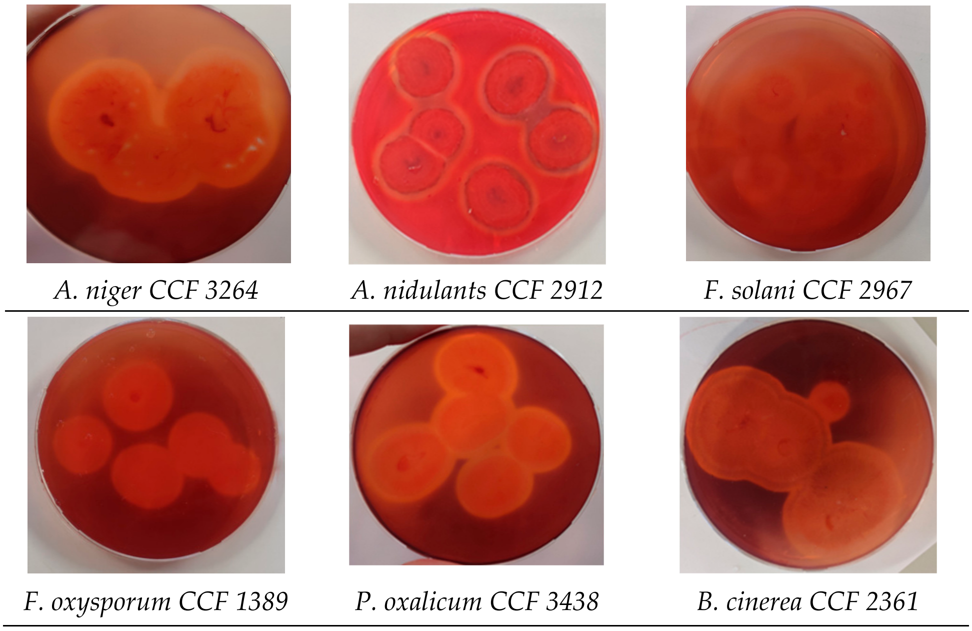

Figure S1. Hydrolytic activity of tested fungal strains on CMC-based agar.

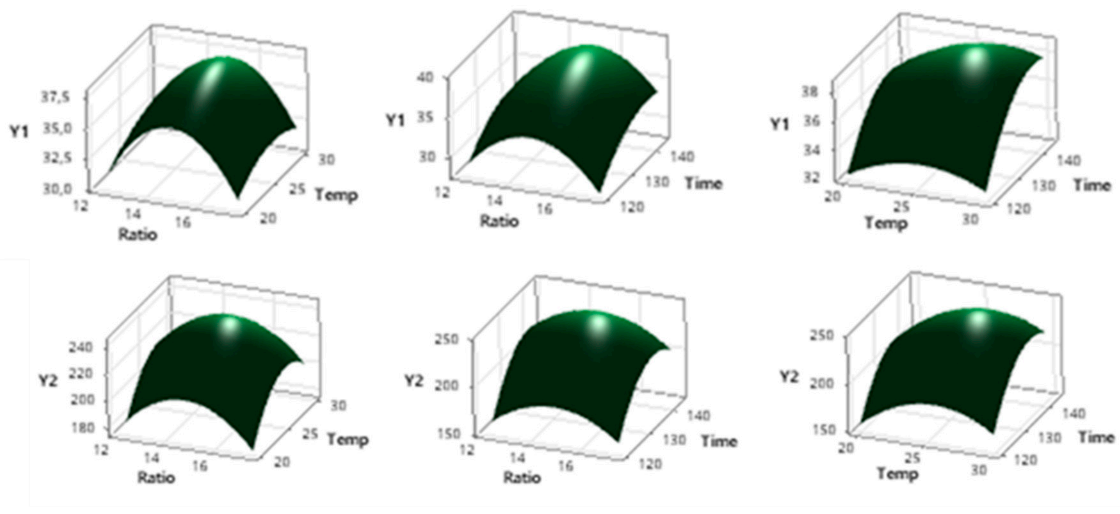

Figure S2. Response surface plots of the interactions between process parameters for RS yield (Y1) and cellulase activity (Y2).

Table S1. Coded coefficients of the mathematical models for Y1 and Y2 responses.

	Model Y1				Model Y2			
**Term**	**Coef**	**SE Coef**	**T-Value**	**P-Value**	**Coef**	**SE Coef**	**T-Value**	**P-Value**
Constant	37.909	0.235	161.25	0.000	6.5285	0.0181	361.65	0.000
Ratio	-0.167	0.216	-0.77	0.458	-0.0601	0.0200	-3.00	0.013
Time	0.527	0.216	2.44	0.035	0.5148	0.0193	26.64	0.000
Temp	2.150	0.216	9.94	0.000	0.2300	0.0193	11.90	0.000
Ratio*Ratio	-5.152	0.412	-12.49	0.000	-0.6164	0.0318	-19.36	0.000
Time*Time	-1.462	0.412	-3.55	0.005	-0.8661	0.0318	-27.21	0.000
Temp*Temp	-1.747	0.412	-4.24	0.002	-0.6651	0.0318	-20.89	0.000
Ratio*Time	-0.010	0.242	-0.04	0.968	-0.1997	0.0232	-8.59	0.000
Ratio*Temp	0.535	0.242	2.21	0.051	0.1961	0.0232	8.44	0.000
Time*Temp	0.440	0.242	1.82	0.099	-0.0823	0.0223	-3.69	0.004

Table S2. ANOVA of mathematical models.

	Mathematical model for Y1				Mathematical model for Y2			
**Term**	**Estimated Value**	**SE**	**T-Value**	**P-Value**	**Estimated Value**	**SE**	**T-Value**	**P-Value**
Const.	37.909	0.235	161.25	0.000	6.5285	0.0181	361.65	0.000
X1	-0.167	0.216	-0.77	0.045	-0.0601	0.0200	-3.00	0.013
X2	2.150	0.216	9.94	0.000	0.5148	0.0193	26.64	0.000
X3	0.527	0.216	2.44	0.035	0.2300	0.0193	11.90	0.000
$X1^2$	-5.152	0.412	-12.49	0.000	-0.6164	0.0318	-19.36	0.000
$X2^2$	-1.747	0.412	-4.24	0.002	-0.8661	0.0318	-27.21	0.000
$X3^2$	-1.462	0.412	-3.55	0.005	-0.6651	0.0318	-20.89	0.000
X1X3	-0.010	0.242	-0.04	0.968	-0.1997	0.0232	-8.59	0.000
X1X2	0.535	0.242	2.21	0.051	0.1961	0.0232	8.44	0.000
X2X3	0.440	0.242	1.82	0.049	-0.0823	0.0223	-3.69	0.004
**R^2**	**R^2_{adj}**		**R^2_{pred}**		**R^2**	**R^2_{adj}**		**R^2_{pred}**
98.59%	97.32%		92.41%		98.89%	97.89%		96.99%
Lack of fit	**F-Value**		**P-Value**		*Lack of fit*	**F-Value**		**P-Value**
	6.81		0.089			53.61		0.985
